# Fatigue in multiple sclerosis: still elusive after all these years

**DOI:** 10.1093/braincomms/fcaf105

**Published:** 2025-03-08

**Authors:** Lauren B Krupp, Kimberly A O’Neill

**Affiliations:** Department of Neurology, NYU Grossman School of Medicine, New York, NY 10016, USA; Department of Neurology, NYU Grossman School of Medicine, New York, NY 10016, USA

## Abstract

This scientific commentary refers to ‘Fatigue in early multiple sclerosis: MRI metrics of neuroinflammation, relapse and neurodegeneration’, by Meijboom *et al.* (https://doi.org/10.1093/braincomms/fcae278).

**This scientific commentary refers to ‘Fatigue in early multiple sclerosis: MRI metrics of neuroinflammation, relapse and neurodegeneration’, by Meijboom *et al.* (https://doi.org/10.1093/braincomms/fcae278).**

The recent study by Meijboom *et al*.^1^ in *Brain Communications* provides a key piece in helping further to understand the puzzle of multiple sclerosis fatigue. The authors performed a comprehensive neuroimaging and clinical assessment of 440 individuals with early multiple sclerosis (within 6 months of the multiple sclerosis diagnosis) who were treatment naïve. They analysed the relation between fatigue and MRI measures of brain and upper cervical cord cross-sectionally and longitudinally. When those with elevated fatigue levels were compared with those with lower fatigue levels, no differences were noted with respect to MRI measures of global and regional white and grey matter volumes, white matter lesion (WML) load and upper cervical spinal cord cross-sectional area Levels C2–3. The authors also assessed new/enlarging WMLs and found no differences between the high and low fatigued groups. These findings can be interpreted as suggesting that fatigue cannot be readily linked to structural brain measures of inflammation or neurodegeneration. The findings are consistent with some prior work that suggested structural correlations of fatigue are not readily identifiable.^2^ In other respects, despite the similar MRI findings between the fatigued and non-fatigued groups, the study was consistent with what we already believed to be true about multiple sclerosis fatigue and about changes in brain MRI in multiple sclerosis. As expected, fatigue was associated with higher depression and disability scores, and the MRI changes among both the high and low fatigue multiple sclerosis subgroups showed that longitudinal decreases in brain volume exceed those of normal ageing.

How to explain this lack of association between fatigue, the most common symptom of multiple sclerosis and one that is intrinsic to the disease, with our most sensitive outcome—brain MRI? Understanding this lack of a structural correlation can be facilitated by recognizing the complex nature of fatigue, which can be defined as a subjective lack of physical and/or mental energy that interferes with daily activities.^3^ Fatigue can be described as transient or persistent, yet many individuals living with multiple sclerosis will face the symptom of fatigue lifelong. Fatigue overlaps with mood disturbances such as depression or anxiety. Nonetheless, many individuals with multiple sclerosis who are without any affective mood disturbance can suffer from severe fatigue.^3^ Other factors that can exacerbate fatigue include sleep disturbance and pain, both common to multiple sclerosis and without clear structural correlations. Some studies using polysomnography have identified sleep abnormalities in patients experiencing fatigue.^4^ Early in the multiple sclerosis disease course, fatigue can interfere in productivity even in the absence of motor or physical impairment. For some, fatigue can occur prior to the diagnosis, and worsening fatigue can accompany multiple sclerosis relapses or occur independently of relapses. Fatigue is very much influenced by psychological factors and has been linked to a sense of loss of control.^5^

Based on its essentially subjective nature, self-report measurement is fundamental in defining the problem. Measures such as the Fatigue Severity Scale^6^ or the Fatigue Impact Scale^7^ were introduced in the late 1980s and early 1990s and remain widely used. While these measures show moderate to strong correlation with one another, they do not fully assess the same aspects of fatigue. More recently, measures introduced by consortia supported by the National Institute of Health (NIH) and other groups have provided short forms and more extensive assessments, e.g. the NIH Patient-Reported Outcomes Measurement System in which shorter and larger item banks can be applied both to multiple sclerosis and other neurologic disorders. Despite the variety of fatigue measures, most show a fair bit of overlap.

One approach in conceptualizing multiple sclerosis fatigue that can be used to interpret the lack of a structural correlation is to recognize that fatigue is both multi-dimensional and multi-factorial. It is likely to be mediated by a host of factors some of which are related to premorbid individual characteristics and others are related to functional consequences of multiple sclerosis including release of cytokines or other signalling molecules and altered brain circuitry or metabolism. Given these complex interactions and the variability across individuals, perhaps it is not surprising that we lack a consistent neuroimaging or circulating biomarker of fatigue.

Changes in white matter tracts have been suggested to contribute to fatigue pathogenesis given their role in psychomotor activity. Whole-brain and regional brain volume loss, as demonstrated in the study by Meijboom *et al*.,^1^ have been less revealing. Nonetheless, a large study of over 4000 participants showed greater WML and low whole-brain volume to be weakly associated with fatigue.^8^ To see such associations, given the relatively weak correlation, large patient samples may be necessary. Other studies have found associations between fatigue with volume loss in the frontal lobes or deep grey structures,^9,10^ but often such findings are not replicated. The role of spinal cord imaging and fatigue is understudied, but the few studies that are available including the paper by Meijboom *et al*.,^1^ have found no significant connections.^3^

There has been quite a bit of interest regarding the role of functional imaging and defining fatigue including abnormalities in both the resting default network and the salience network. While some functional imaging studies have distinguished multiple sclerosis individuals with fatigue from those without fatigue,^11^ the findings have not been reliably reproduced by other groups. There is hope that advancing analytic techniques of white matter integrity or metabolomic changes may pick up subtle neuroimaging correlations that have not been previously identified. To better understand the pathophysiology and neuroimaging underpinnings of fatigue, additional research with large datasets will be necessary.

Despite the absence of reliable fatigue biomarkers, fatigue can be managed in many individuals with multiple sclerosis. In general, medications are not more effective than placebo but non-pharmacologic interventions including mindfulness, yoga and cognitive behavioural therapy can led to symptomatic improvement in multiple sclerosis fatigue.^3^

The study by Meijboom *et al*.^1^ is a helpful addition to our understanding of fatigue and reminds us of the complexity and heterogeneity of this subjective experience.

## Data Availability

No new data were generated or analysed in support of this commentary.
